# Surface Markers for Chondrogenic Determination: A Highlight of Synovium-Derived Stem Cells

**DOI:** 10.3390/cells1041107

**Published:** 2012-11-16

**Authors:** Doug D. Campbell, Ming Pei

**Affiliations:** 1 Stem Cell and Tissue Engineering Laboratory, Department of Orthopaedics, West Virginia University, Morgantown 26506, WV, USA; Email: ddcampbell88@gmail.com; 2 Division of Exercise Physiology, West Virginia University, Morgantown 26506, WV, USA; 3 Mechanical and Aerospace Engineering, West Virginia University, Morgantown 26506, WV, USA

**Keywords:** synovium-derived stem cell, mesenchymal stem cell, cartilage regeneration, surface phenotype, chondrogenic potential

## Abstract

Cartilage tissue engineering is a promising field in regenerative medicine that can provide substantial relief to people suffering from degenerative cartilage disease. Current research shows the greatest chondrogenic potential for healthy articular cartilage growth with minimal hypertrophic differentiation to be from mesenchymal stem cells (MSCs) of synovial origin. These stem cells have the capacity for differentiation into multiple cell lineages related to mesenchymal tissue; however, evidence exists for cell surface markers that specify a greater potential for chondrogenesis than other differentiation fates. This review will examine relevant literature to summarize the chondrogenic differentiation capacities of tested synovium-derived stem cell (SDSC) surface markers, along with a discussion about various other markers that may hold potential, yet require further investigation. With this information, a potential clinical benefit exists to develop a screening system for SDSCs that will produce the healthiest articular cartilage possible.

## 1. Introduction

Healthy, pain-free joints are a luxury that most people fail to appreciate. It is not until destruction has set in and rendered the joint non-functioning that one realizes how crucial healthy joints are for their simple, everyday movement. Connective tissue destruction may occur from acute injuries or chronic causes, like osteoarthritis (OA) and rheumatoid arthritis (RA). Arthritis is a common disease, diagnosed in one in five American adults, and is to blame for causing severe impairment in the quality of life for the elderly due to pain, inflammation, and joint degradation [1]. As one of the leading causes of disability, arthritis limits daily activities for over seven million American adults [2]. Unfortunately, due to the avascular nature of cartilage, the body has virtually no ability to repair the site of damage; untreated problems lead to the progression of OA, currently the most common form of arthritis [3,4]. 

Current treatments generally involve managing pain and inflammation through lifestyle modifications in order to slow the progression of OA. Recommendations include avoiding high-impact activities, losing weight, using non-steroidal anti-inflammatory medications, and physical therapy. These treatments only mask the problem. Autologous chondrocyte transplantation has provided an effective method of durable repair in a majority of patients undergoing the procedure. Recent reports suggest that the clinical and functional outcomes remain high even 10–20 years after implantation [5] and the quality of the repair tissue is similar to the surrounding normal cartilage [6]. However, limitations exist due to donor-site availability, limited expandability, and dedifferentiation of chondrocytes [7,8,9]. In order to truly heal these patients, the damaged articular cartilage must be regenerated to prevent any further symptoms.

Research has begun to address the idea of tissue regeneration as a form of treatment for degenerative joint diseases. At the forefront of treatment options are a family of stem cells, known as mesenchymal stem cells (MSCs), which hold the potential for differentiation into multiple lineages, such as bone, fat, cartilage, tendon and ligament, muscle, and marrow stroma [10,11]. Since the beginning of this field of research, MSCs have been isolated from a variety of sources, including bone marrow, adipose, periosteum, muscle, perichondrium, and synovium [12,13,14,15,16,17,18]. Along with their multipotent capabilities, MSCs also display an amazing ability to self-replicate [19]. It has been shown that MSCs of synovial origin have a higher capability for chondrogenesis when compared to MSCs from other origins [20]. This observation suggests the possibility of a tissue-specific stem cell that can be harvested from the synovium that will preferentially differentiate into articular cartilage [21,22]. 

While MSCs lack a clearly defined surface marker profile, they do present an array of cell surface markers that provide clues to the signaling interactions these cells undergo, especially with regard to proliferation and differentiation [23,24]. The Mesenchymal and Tissue Stem Cell Committee of the International Society for Cellular Therapy provides minimum defining criteria for MSCs. These criteria include the requirement for plastic adherence, as well as positive expression of the surface markers CD73, CD90, and CD105 and negative expression of CD45, CD34, CD14 or CD11b, CD79α or CD19, and HLA-DR surface markers [25]. 

To date, many surface markers have been characterized from bone marrow derived stem cells (BMSCs). It has been proven that no significant differences exist between synovium derived stem cell (SDSC) surface markers and BMSC surface markers [26]. Compared to SDSCs as a tissue-specific stem cell for chondrogenesis [20], BMSCs tend to proceed to endochondral bone formation when incubated in a chondrogenic induction medium, indicating these cells have a bias toward osteogenic lineage differentiation (Table 1). Current SDSC markers may not be specific to the synovium, yet they may still provide clues to the chondrogenic potential held by these cells. While the mechanisms of these cell surface markers with respect to chondrogenesis may not be entirely understood, they still may provide insight for cartilage engineering.

**Table 1:** Difference in cell properties between synovium-derived stem cell (SDSCs) and bone marrow derived stem cells (BMSCs)

Properties	SDSCs	BMSCs
Genetic Profile	Gene profile of intraarticular tissue MSCs (such as SDSCs) and chondrocytes were closer to each other [27]	Gene profile of intraarticular tissue were a closer match than extraarticular tissue MSCs (such as BMSCs) [27]
	A significant 5- to 10-fold reduction in the osteogenic marker levels compared to BMSCs [26]	A significant 5- to 10-fold increase in the osteogenic marker levels compared to SDSCs [26]
	Lower levels of Activin A than BMSCs, probably related to less hypertrophy [26]	Higher levels of Activin A than SDSCs, probably indicative of hypertrophic nature [26]
Proliferation Capacity	Colony-forming potential per nucleated cell = 1 in 12.5–80 [28]	Colony-forming potential per nucleated cell = 1 in 10^3^–10^4^[28]
	Age-independent [15]	Age-dependent [29]
Differentiation Potential	SDSCs expanded on DSCM exhibited not only an enhanced chondrogenic potential but also a decreased osteogenic capacity [30] For full-thickness osteochondral	BMSCs expanded on DSCM exhibited not only an enhanced chondrogenic potential but also a concomitant increase in osteogenic capacity [32]
	defects in a rabbit model, SDSC implantation retained metachromatic staining in repaired cartilage up to 24 weeks and did not show signs of ossification [31]	Although *in vivo* repair by BMSCs resulted in clinically acceptable outcomes, the repaired tissue had a cartilaginous-to-fibrous appearance, perhaps indicating inferior mechanical properties of the tissue compared with native tissue [33]

Utilizing the possibility of a tissue-specific stem cell, this review hopes to present evidence for SDSC surface markers that can signal an increased potential for cartilage regeneration. This review will present known MSC phenotypic surface markers, many of which have been discussed in the 2008 review “Mesenchymal Stem Cells and Their Surface Receptors”, by Docheva *et al.* [10]. However, an emphasis will be placed on markers for SDSCs that are detectable *via* flow cytometry and signal an enhanced chondrogenic potential (Table 2), in order to maximize the clinical effectiveness of cartilage engineering and regeneration.

**Table 2:** A summary of potential chondrogenic surface markers for SDSCs.

A: Chondrogenic Surface Markers for SDSCs	
CD73	High expression of *SOX9* and *ACAN* with concomitant presence of *COL10A1* during chondrogenic induction [34]
CD90	Presence in 99.98% of dedifferentiated articular chondrocytes [35]; positively correlated with SDSC chondrogenic potential [36]; and increased presence in DSCM-expanded SDSCs [30]
CD105	Presence of type II collagen and aggrecan increased from day 0-28 of chondrogenic induction, *SOX9* present throughout culturing period [37]; and CD105+ human adipose stem cells displayed an enhanced chondrogenic potential [38]
CD106	Minimal expression of *COL2A1* and *ACAN* [34]; and high expression of *COL10A1* during differentiation [34]
CD166	Almost exclusive zonal distribution within superficial and middle zones of articular cartilage [39]; and 99% co-expression with CD105 [39]
CD271	Very high levels of type II collagen and aggrecan at 28 days of chondrogenic induction and stable presence of *COL10A1* during differentiation [34]
B: Alternative Chondrogenic Surface Markers for SDSCs	
CD29	High CD29 expression BMSCs display greater chondrogenic differentiation capacity than low CD29 expression BMSCs [40]
CD44	Highly expressed in chondrogenic subpopulation from nasal septal cartilage zone [41]
CD56	Expressed in highly clonogenic population of CD271+ BMSCs [42]; and marker for chondrocytes with enhanced ability for pellet formation when utilized in conjunction with mesenchymal stem cell antigen-1 [43]
GD2	Co-expressed in 95% of BMSCs with CD45-/CD105+/CD73+ [44]; and CD271+ BMSCs co-express GD2 [44]
3G5	Highly positive staining in CD13+/CD44+/CD90+/CD105+ BMSCs [45]; and express elevated GAG content, *COL2A1*, *COL1A2*, and *SOX6* when cultured in a hypoxic environment with chondrogenic medium [45,46]
SSEA-4	SSEA-4+/CD45- subpopulation of BMSCs exhibit superior growth and maintenance of multipotent capabilities [47]; identifying proliferative cell subpopulations that have increased chondrogenic potential [48]; and increased expression in DSCM-expanded BMSCs [32]
Integrins α2 and β5	Upregulated expression in DSCM-expanded BMSCs [32]
Integrins α10	Expression decreases on BMSCs over prolonged monolayer culturing; α10+ BMSCs, treated with FGF-2, display upregulation of *SOX9* [49]
Dlk1/Pref-1/FA1	*DLK1* expresses similar patterns to *SOX9* and *COL2A1* during chondrogenesis in mouse limb bud cells [50]; downregulation of *DLK1* in the presence of TGF-β1 [50]; DLK1/FA1 marks transition from proliferating chondrocytes to pre-hypertrophic chondrocytes in mouse embryonic limb development [51]
Notch	Implicated in regulation of cartilage and bone development [52]; loss of notch signaling in mouse limb MSC results in delay in onset of chondrocyte maturation and reduction in chondrocyte proliferation [53]; and *in vitro* analysis shows increased *COL2A1* and decreased *COL10A1* in chondrocytes with blocked notch signaling [52]
Sca-1	Sca-1- cells in a chondrogenic medium display intense Safranin-O staining [54]
ALDH	ALDH-bright subpopulations are highly enriched with cells expressing stem cell surface markers [55]; broad superfamily with multiple isozymes involved in various stem cell lineages, as well as cell proliferation and differentiation [56]; and ALDH-enriched adipocyte stem cells display no enhanced chondrogenic potential [57]

## 2. Chondrogenic Surface Markers for SDSCs

One of the first experiments to examine the chondrogenic potential of specific SDSC cell markers was conducted by Jo *et al.* in 2007 [28]. This lab demonstrated that cells from the synovium contained a stem cell population with a specific cell surface characterization that was capable of undergoing chondrogenesis. While investigating synovial cells obtained from OA patients, it was found that cells that were immediately isolated were positive for CD10, CD13, CD14, CD34, CD44, CD45, CD49a, CD62e, CD73, and HLA-DR. After the first passage, the hematopoeitic stem cell markers CD14, CD34, and CD45, as well as CD62e and HLA-DR disappeared. CD105 and CD166 appeared after this first passage, along with an increased expression of CD10, CD13, CD44, CD49e, and CD73. Subsequent passages showed no variance in surface markers, yet the chondrogenic differentiation potential still remained. This experiment was one of the first examples to show that certain SDSC surface markers appeared to be related to the chondrogenic process. The fact that the synovial samples came from patients with degenerative arthritis may factor into the initial immunophenotype of the cells; nevertheless, Jo *et al.* provided important information to guide future projects in the field of chondrogenic surface markers. 

At passage 1, CD73+ cells had the highest expression of *SOX9* and *ACAN*, as well as the second highest expression of *COL2A1* at day 46 of chondrogenic induction when subsequently compared with CD106+ and CD271+ subpopulations. The elevated levels of *SOX9* suggested a great potential for SDSC chondrogenesis, but further research is required to understand why their production of type II collagen was lacking in a long-term culture compared to CD271+ cells. The CD73+ cells also showed a stable presence of *COL10A1*, suggesting the presence of hypertrophy during differentiation [34].

CD90 may also play a role in chondrogenesis with evidence presented in a study performed by Diaz-Romero *et al.* Human articular chondrocytes were cultured in monolayer for dedifferentiation. Flow cytometry was performed with the differentiated chondrocytes and two weeks into dedifferentiation in order to compare the appearance of surface markers. CD90 was found to appear in 99.98% of all dedifferentiated articular chondrocytes, suggesting that this marker may have a key role in differentiation of MSCs into cartilage [35]. Furthermore, Nagase *et al.* found that CD90 is an important indicator of the chondrogenic differentiation potential of SDSCs [36]. It has also been observed that SDSCs expanded on decellularized stem cell matrix (DSCM) acquired an enhanced proliferation and chondrogenic differentiation potential; interestingly, CD90+ cells increased concurrently in this population [30]. Further investigations must be carried out in order to determine if CD90 is truly related to chondrogenesis or simply a surface marker for undifferentiated MSCs.

CD105+ cells were isolated using a magnetic separator and cultured using a transforming growth factor-β3 (TGF-β3) medium in order to induce differentiation toward chondrocyte-like cells. The subsequent spheroids were collected after 14, 28, and 46 days and stained in order to verify the presence of type I collagen, type II collagen, and aggrecan. Analysis revealed cells that shared similar characteristics to chondrocytes due to their positive staining for Safranin-O and Alcian blue throughout the experiment. Immunohistochemical analysis showed that, in this subpopulation, the presence of type II collagen and aggrecan increased throughout the culturing period up until day 28, after which it then began to decline. Real-time polymerase chain reaction (RT-PCR) results supported this analysis and showed the presence of *SOX9* throughout the experiment. In addition to confirming the chondrogenic capacity of CD105+ SDSCs, no significant differences were noted between subpopulations from normal and OA synovial membrane samples [37]. In human adipose stem cell studies, CD105+ sorted cells displayed an enhanced chondrogenic potential compared to the CD105- subpopulation, with stronger type II collagen staining and a higher expression of *COL2A1* and *ACAN* genes. These cells were also capable of forming into a homogenous cartilage-like tissue when cultured in a chondrogenic medium on a scaffold [38]. This evidence, when taken together, may promote the benefits of utilizing CD105 as a chondrogenic surface marker for SDSCs.

CD106+ cells showed weak differentiation into chondrocyte-like cells, indicated by their minimal expression of *COL2A1* and *ACAN*, along with high expression of *COL10A1*. Arufe *et al.* suggested that perhaps CD106+ cells are less differentiated than CD73+ and CD271+ cells and therefore have an absence of chondrogenic capability in comparison [34].

CD166, a central mediator in cell adhesion, growth, and migration, has been suggested as a biomarker for the identification and localization of mesenchymal progenitor cells in human articular cartilage with a high chondrogenic potential [39,58]. One study investigated the zonal distribution of CD166+ chondrocytes in articular cartilage, where it was discovered that these chondrocytes were almost exclusively located in the superficial and middle zones [39], areas known to be important in articular cartilage growth in young animals [39,59]. Interestingly, 99% of the mesenchymal progenitor cells in this study co-expressed CD105 and CD166 [39]. 

CD271+ cells showed a great capability for adipogenic and chondrogenic differentiation, according to Arufe *et al.*, as well as very high levels of type II collagen and aggrecan staining at day 28. It is worth mentioning that no significant difference in expression of *SOX9* was found between the CD106+ and CD271+ subpopulations. This CD271+ subpopulation of cells also showed a stable, yet elevated presence of *COL10A1*, suggesting that hypertrophy had taken place during differentiation [34]. 

With these data, it is important to note that the surface cell markers are not appearing in isolation. An additional component of the Arufe *et al.* 2010 paper was their inclusion of data comparing the frequencies of cell surface markers present on their synovial membrane MSC subpopulations [34]. In the CD73+ subpopulation, they found that more than 50% of the cells were also positive for CD44, CD69, and CD90, while less than 40% were positive for CD105. In the CD106+ subpopulation, more than 60% of the cells were positive for all of the tested markers. In the CD271+ subpopulation, more than 80% of the cells were also positive for CD69, CD90, and CD105 with less than 40% positive for CD44 and CD73. These data suggest that CD105 may be a key indicator for chondrogenic potential, due to its high expression in the CD271+ subpopulation which exhibited the greatest type II collagen and aggrecan staining. The appearance of all surface markers tested on the CD106+ subpopulation may support the notion of these cells being less differentiated, as previously mentioned [34]. Future research may discover that the relationships between surface markers are just as important as the presence of a single marker when determining a population’s differentiation potential.

## 3. Alternative Markers for Further Investigation

Alternate surface markers may be present that will provide a unique way of identifying MSCs. While few, if any, of these markers have been examined in relation to SDSCs and chondrogenesis, we believe that they still warrant mention for possible future investigation.

CD29 may be potentially useful in SDSCs due to experimental evidence seen with BMSCs. After culture expansion for a total of 7–9 passages, CD105+ BMSCs were further divided into groups with high expression levels of CD29 and low expression levels of CD29, and then tested with osteogenic, chondrogenic, and adipogenic differentiation. A similar differentiation potential was noted between the osteogenic and adipogenic studies; however, chondrogenic differentiation showed a significant difference between the subpopulations. After incubation in chondrogenic induction medium [chemically defined Dulbecco’s Modified Eagle Medium (DMEM) with 1 ng/mL of recombinant human TGF-β3], the high CD29 expression group displayed a greater chondrogenic differentiation capacity than the low CD29 expression group, while the low expression subpopulation showed no difference compared to the control (DMEM with 20% fetal bovine serum). This result was verified with immunohistochemical analysis of type II collagen, as well as RT-PCR analysis of chondrocyte-specific genes [40]. The relationship between chondrogenic potential and CD29 expression is evident in BMSCs; however, the relationship with CD29 and SDSCs remains uncertain. 

While CD44 was previously mentioned as a marker for SDSCs, no studies have been conducted to date to test its chondrogenic potential. Recently, a study was conducted to isolate and characterize a chondrogenic subpopulation from the human nasal septal cartilage zone. This niche was hypothesized to provide potent chondrogenic progenitor cells due its similarities to articular cartilage niches. The nasoseptal cells showed a constitutive expression of the *SOX9* transcription factor, with the ability to produce large amounts of sulfated glycosaminoglycans and type II collagen in a medium without any chondrogenic growth factors (TGF-βs and bone morphogenetic proteins). These progenitor cells at passage 1 were found to be positive for CD44, CD73, and CD105, as well as negative for CD146. CD44, in particular, was found to be highly expressed in this subpopulation of cells [41]. In addition to supporting the roles of CD73 and CD105 in chondrogenesis, this study provides evidence that CD44 may also hold a key role, although specifics in relation to SDSCs need further investigation.

CD56, best known as a marker for natural killer, neural, and muscle cells, has an epitope that has been discovered to be expressed on a highly clonogenic population of CD271-positive BMSCs [42,43]. This marker, when utilized in conjunction with mesenchymal stem cell antigen-1 for sorting, produced viable chondrocytes with the capability to produce pellets close to five times greater in size than mesenchymal stem cell antigen-1 CD56- sorted cells [43].

GD2, the ganglioside marker found largely in neurons, may also be a marker of importance [60]. Glycolipids have been utilized as excellent biomarkers for stem cells due to their location on the outer leaflet of the plasma membrane [61]. GD2+ BMSCs sorted via immunomagnetic beads displayed typical MSC morphology and plastic adherence and maintained the ability to differentiate into chondroblasts, osteoblasts, and adipocytes. It was also discovered that 95% of BMSCs with the surface phenotype CD45-/CD105+/CD73+ also expressed GD2. CD271+ BMSCs have also been found to coexpress GD2 [44]. Due to previous results indicating the enhanced chondrogenic potential of CD73+, CD105+, and CD271+ subpopulations, GD2 may be a single surface marker capable of indicating MSCs with an enhanced chondrogenic potential.

Another cell surface ganglioside worth investigating may be recognized by the antibody 3G5, originally used as a marker for vascular pericytes. It was hypothesized that MSCs in bone marrow may originate from pericytes, which have been shown to be able to differentiate into osteoblasts, chondrocytes, and adipocytes [62]. This marker may be a potentially useful chondrogenic marker, due to its high positive staining in a subpopulation of BMSCs at passage 2, concurrently expressing strong staining for CD13, CD44, CD90, and CD105. These 3G5+ cells successfully differentiated into chondrocytes when cultured in a chondrogenic medium, with an even greater response being seen after placement in a known chondrogenic-stimulating hypoxic environment [45,46]. Such chondrogenic changes included an increased glycosaminoglycan content, elevated *COL2A1* and *COL11A2*, as well as SOX6 activity [46]. This evidence suggests 3G5 may play an important role in chondrogenesis; however, its mechanisms in SDSCs require further investigation.

The glycolipid SSEA-4 may also be a marker of interest. This stage-specific embryonic antigen has been used in the past for embryonic stem cell identification and may also be used to identify MSCs [61]. At passage 2, SSEA-4+/CD45- cell subpopulations from bone marrow exhibited superior growth and maintained their multipotent capabilities compared to unsorted MSCs [47]. The cell surface marker SSEA-4 may play a role in identifying proliferative cell subpopulations that have increased chondrogenic potential [48]. In DSCM-expanded passage 5 human BMSCs, an increased expression of SSEA-4 was observed, along with a superior ability for cartilage formation and an enhanced osteogenic potential [32]. 

In the study mentioned just prior, DSCM-expanded passage 5 BMSCs also expressed enhanced levels of integrins α2 and β5, with a decrease in integrin α5 [32]. The osteopontin receptor integrin β5, known to be constantly expressed in BMSCs, is thought to be involved in cellular migration and proliferation. It was recently suggested that it may have a role in chondrogenic differentiation [63]. The upregulation of integrins α2 and β5 may play a role in the enhanced chondrogenesis seen in DSCM-expanded BMSCs [32]; further investigation into their possible roles in chondrogenesis is warranted.

When MSCs from bone marrow were cultured in monolayer, these cells expressed the integrin subunits α10 and α11. Over prolonged culturing, α10 expression declined, suggesting that this integrin subunit signaled undifferentiated, young cells. This finding was further supported by the upregulation of α10 when the MSCs were treated with the proliferative agent fibroblast growth factor-2 (FGF-2). These FGF-2-treated α10-expressing cells were found to have a higher chondrogenic potential due to their concurrent upregulation of *SOX9*. Interestingly, integrin subunits α1 and α11 decrease during this time period [49]. This evidence suggests that integrin subunits, especially α10, may be indicative of chondrogenic potential, while others may signal a lack of bias toward chondrogenesis.

Delta like-1/preadipocyte factor-1/fetal antigen-1 (Dlk1/Pref-1/FA1) is a transmembrane protein, originally identified as a negative regulator of adipocyte differentiation; it has recently been discovered as a possible surface marker for chondroprogenitor cells. In a study with mouse limb bud cells, during the chondrogenic process, RT-PCR analysis shows that *DLK1* expressed similar patterns to known chondrogenic markers *SOX9* and *COL2A1*. It was also found that TGF-β1, a known inducer of chondrogenesis, appears to be a possible regulator of *DLK1* expression. TGF-β1 treated limb bud cells resulted in chondrocytes that maintained their proliferative stage, while inhibiting mineralization and maturation. These cells also displayed a downregulation of *DLK1*, indicating that important communication must take place between TGF-β1 signaling pathways and DLK1/FA1 during early chondrogenesis [50]. It was also shown that during mouse embryonic limb development, along with human embryonic stem cell-derived teratoma models, the appearance of DLK1/FA1 marked the transition from immaturely proliferating chondrocytes to pre-hypertrophic chondrocytes [51].

The Notch signaling pathway may serve as a useful chondrogenic marker, due to its important role in regulating cell proliferation, differentiation, fate determination, stem cell self-renewal, and cell-cell communication [52]. The Notch pathway surface receptor responds to multiple ligands, one of which is the previously mentioned Delta-like-1 ligand [53]. The Notch intra-cellular domain (NICD), which translocates to the nucleus to interact with transcriptional elements upon signaling, is believed to be involved in two paths: one that is dependent on the transcriptional regulator RBPjκ, and one that is independent. A study by Kohn *et al.* provided data suggesting that chondrocyte maturation is mediated by the RBPjκ-dependent pathway, while the RBPjκ-independent pathway is involved in the regulation of chondrocyte proliferation and columnar chondrocyte organization. While the exact mechanisms of the RBPjκ-independent signaling pathway is not known in mammalian cells, the NICD has recently been shown to interact with members of BMP, TGF-β, and WNT/β-Catenin; all are involved in the regulation of cartilage and bone development [52]. Hilton *et al.* provided genetic evidence for the importance of Notch signaling in embryonic chondrogenesis by removing various upstream Notch signaling components from mouse limb MSCs and investigating the outcome in cartilage development. In this experiment, the loss of Notch signaling resulted in a delay in the onset of chondrocyte maturation, as well as a reduction in chondrocyte proliferation [53]. *In vitro* studies confirmed the delay in chondrocyte maturation with a loss of Notch signaling. Two populations of primary chondrocytes were plated in monolayer; one was treated with the Notch signal inhibitor DAPT and a control had no DAPT. Maturation was assessed at seven days, where it was discovered that DAPT-treated chondrocytes displayed significantly increased *COL2A1* compared to controls, as well as significantly decreased *COL10A1* [52]. These studies provide evidence for the involvement of Notch signaling in proliferation and maturation of chondrocytes; however, the role of Notch in SDSC chondrogenesis remains unknown, but seems promising as a potential surface marker.

Stem cell antigen-1 (Sca-1) may potentially serve as a negative marker for enhanced chondrogenesis. Utilizing cells from mouse calvaria, Sca-1+ and Sca-1- subpopulations were examined to determine their multipotent differentiation capabilities. The Sca-1+ cell population displayed an enhanced adipogenic capability while, more interestingly, Sca-1- cells were able to differentiate into bone and cartilage. Sca-1- cells, cultured in a chondrogenic growth medium of TGF-β1, displayed intense Safranin-O staining, signaling a strong ability to produce type II collagen. This ability to differentiate into cartilage remained until passage 6, suggesting a potent chondrogenic capability for these Sca-1- cells [54]. 

Moving away from markers on the cell surface, it may be possible to isolate chondrogenic SDSCs via the intracellular enzyme aldehyde dehydrogenase (ALDH), utilizing Aldefluor positivity imaging. This enzyme is highly expressed in multiple adult stem cell lineages, as well as progenitor cell lineages, serving as a marker of stemness [55]. The human ALDH superfamily has been found to have 19 functional genes, categorized in 11 different families and 4 additional subfamilies, providing multiple isozymes for functional analysis. *ALDH1A1*, first indicated as a marker for hematopoetic progenitor cells isolated from bone marrow and neural stem cells, can be utilized as a marker identifying normal or cancer stem cells. *ALDH1A1*, along with *ALDH3A1*, are known to be involved in cell proliferation and differentiation [56]. It was recently discovered that ALDH-bright subpopulations are highly enriched with cells that express stem cell surface markers, such as CD34, CD117, CD105, CD127, CD133, and CD166. These cells also displayed *in vitro* activities characteristic of stem and progenitor multi-lineage hematopoietic progenitor cells, endothelial progenitor cells, and most importantly for this review, progenitors that eventually gave rise to multipotent mesenchymal stem cells [55]. Interestingly, in a study investigating human adipose stem cells, it was found that ALDH-enriched cells displayed no enhanced chondrogenic potential in comparison to unsorted cells when expanded to passage 9 [57]. Gene profile analysis of MSCs from different tissue sources showed that *ALDH1A1* is downregulated in MSCs *versus* differentiated fibroblasts [64]. Investigation into the chondrogenic potential of ALDH-enriched SDSCs may uncover a unique marker for chondrogenesis.

## 4. Conclusion

The results of current work on MSCs with bone marrow and synovial origins indicate that surface markers may indicate an enhanced potential for chondrogenesis. To date, research supports CD105 as potentially the most useful marker for SDSC isolation with differentiation into cartilage; however, CD73, CD90, and CD271 may also provide utility. Further investigations must be conducted to determine if these markers hold more potential in isolation or in conjunction with one another, especially with regard to MSCs of synovial origin. Novel markers may also still exist; however, they may only provide markers for undifferentiated MSCs, rather than chondrogenic progenitor cells.
